# Quantification of one Prenylated Flavanone from *Eysenhardtia platycarpa* and four derivatives in Ex Vivo Human Skin Permeation Samples Applying a Validated HPLC Method

**DOI:** 10.3390/biom10060889

**Published:** 2020-06-10

**Authors:** Paola Bustos-Salgado, Berenice Andrade-Carrera, María Luisa Garduño-Ramírez, Helen Alvarado, Ana Calpena-Campmany

**Affiliations:** 1 Department of Pharmacy and Pharmaceutical Technology and Physical Chemistry, Faculty of Pharmacy and Food Science, University of Barcelona, Joan XXIII Av. 29–31, 08028 Barcelona, Spain; pbustosa19@alumnes.ub.edu (P.B.-S.); hl_alvarado@ub.edu (H.A.); 2 Facultad de Ciencias Química e Ingeniería, Universidad Autónoma del Estado de Morelos, Av. Universidad 1001, Cuernavaca 62209, Morelos, Mexico; bereniceac@uaem.mx; 3 Centro de Investigaciones Químicas, Instituto de Investigación en Ciencias Básicas y Aplicadas, Universidad Autónoma del Estado de Morelos, Av. Universidad 1001 Cuernavaca, Cuernavaca 62209, Morelos, Mexico; lgarduno@ciq.uaem.mx; 4 Institute of Nanoscience and Nanotechnology (IN2UB), University of Barcelona, 08007 Barcelona, Spain

**Keywords:** prenylated flavanones, HPLC, ex vivo permeation, bioanalytical method validation

## Abstract

Prenylated flavanones are polyphenols that have diverse biological properties. The present paper focuses on a HPLC method validation for the quantification of prenylated flavanones (2*S*)-5,7-dihydroxy-6-(3-methyl-2-buten-1-yl)-2-phenyl-2,3-dihydro-4H-1Benzopyran-4-one **1** and derivatives (2*S*)-5,7-bis(acetyloxy)-6-(3-methyl-2-buten-1-yl)-2-phenyl-2,3-dihydro-4H-1-Benzopyran-4-one **A**; (2*S*)-5-hydroxy-7-methoxy-6-(3-methyl-2-buten-1-yl)-2-phenyl-2,3-dihydro-4H-1-Benzopyran-4-one **B**; (8*S*)-5-hydroxy-2,2-dimethyl-8-phenyl-3,4,7,8-tetrahydro-2H,6H-Benzo[1,2-b:5,4-bˈ]dipyran-6-one **C**; and (8*S*)-5-hydroxy-2,2-dimethyl-8-phenyl-7,8-dihydro-2H,6H-Benzo[1,2-b:5,4-bˈ]dipyran-6-one **D** applied in biopharmaceutic studies. The linear relationships are proven with significant correlation coefficients (R^2^ ˃ 0.999) in the range of 1.56 to 200 μg/mL with low limits of detection and quantification, on average of 0.4 μg/mL and 1.2 μg/mL, respectively. The validation method used in this work is highly accurate and precise, with values lower than 15%. The relative standard deviation values of repeatability of the instrumental system are demonstrated with less than 0.6% for all studied flavanones. Therefore, the applicability method of the quantification of the prenylated flavanones was established using the permeation of human skin in the Franz cell system. During the method previously described, there was no interference observed from human skin components in ex vivo permeation studies.

## 1. Introduction

Prenylated flavanones are part of a diverse class of natural flavonoids conformed of oxygen-containing heterocycles and prenyl substituents. They have proven to have antibacterial and anti-fungal properties conferring protection to plants against diseases [1,2,3,4]. Nowadays, several studies have confirmed their biological effects against oxidation, obesity, and inflammation, which may be useful in the prevention of several diseases, including cancer [5,6,7].

**Figure 1:**
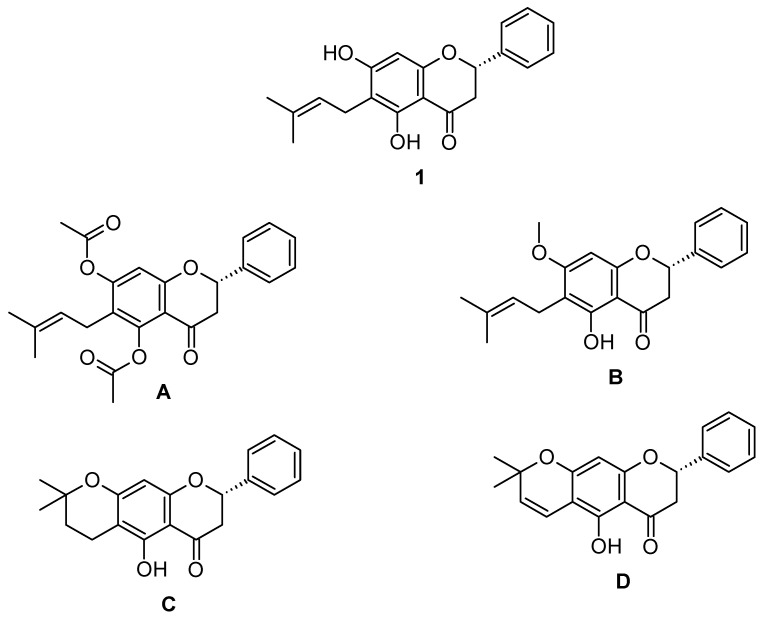
Chemical structures of studied compounds. (2*S*)-5,7-dihydroxy-6-(3-methyl-2-buten-1-yl)-2-phenyl-2,3-dihydro-4H-1Benzopyran-4-one (**1**); (2*S*)-5,7-bis(acetyloxy)-6-(3-methyl-2-buten-1-yl)-2-phenyl-2,3-dihydro-4*H*-1-Benzopyran-4-one (**A**); (2*S*)-5-hydroxy-7-methoxy-6-(3-methyl-2-buten-1-yl)-2-phenyl-2,3-dihydro-4*H*-1-Benzopyran-4-one (**B**); (8*S*)-5-hydroxy-2,2-dimethyl-8-phenyl-3,4,7,8-tetrahydro-2*H*,6*H*-Benzo[1,2-*b*:5,4-*bˈ*]dipyran-6-one (**C**); and (8*S*)-5-hydroxy-2,2-dimethyl-8-phenyl-7,8-dihydro-2*H*,6*H*-Benzo[1,2-*b*:5,4-*bˈ*]dipyran-6-one (**D**).

The genus *Eysenhardtia* comprises 14 species, and some of them, including *E. platycarpa,* which contains prenylated flavanones, are widely used in traditional Mexican medicine for the treatment of kidney and bladder infections [8]. *E. platycarpa* is a tree found throughout southern Mexico and is popularly known as “taray”, “palo dulce” and “palo azul”. Previous studies on *Eysenhardtia* species have highlighted hypoglycemic and antidiabetic properties [9]. The antioxidant activity of the alcoholic extract of *E. platycarpa* was also confirmed [10,11]. Domínguez-Villegas et al. isolated five flavanones from a methanolic extract obtained from the aerial parts of *E. platycarpa*, one of them were (2*S*)-5,7-dihydroxy-6-(3-methyl-2-buten-1-yl)-2-phenyl-2,3-dihydro-4H-1Benzopyran-4-one **1** (Figure 1). The formerly mentioned compounds proved to have anti-inflammatory activity. Furthermore, flavanones revealed a high percentage reduction of free radical DPPH (2,2-Diphenyl-1-picrylhydrazyl), and thus exhibited strong cytotoxic activity on brine shrimp [12].

In addition, the prenylated flavanone **1**, as well as its derivatives obtained from structural modifications: (2*S*)-5,7-bis(acetyloxy)-6-(3-methyl-2-buten-1-yl)-2-phenyl-2,3-dihydro-4*H*-1-Benzopyran-4-one **A**; (2*S*)-5-hydroxy-7-methoxy-6-(3-methyl-2-buten-1-yl)-2-phenyl-2,3-dihydro-4*H*-1-Benzopyran-4-one **B**; (8*S*)-5-hydroxy-2,2-dimethyl-8-phenyl-3,4,7,8-tetrahydro-2*H*,6*H*-Benzo[1,2-*b*:5,4-*bˈ*]dipyran-6-one **C**; and (8*S*)-5-hydroxy-2,2-dimethyl-8-phenyl-7,8-dihydro-2*H*,6*H*-Benzo[1,2-*b*:5,4-*bˈ*]dipyran-6-one **D** (Figure 1); were loaded into polymeric nanoparticles exhibiting cytotoxic potential against pancreatic cell line MiaPaCa-2 [13].

The increasing interest in the medicinal properties of flavanones, with specific anti-inflammatory activity on skin diseases [14], has led to a demand for accurate, reproducible, and sensitive analytical methods to quantify new compounds that have not been validated yet. High-performance liquid chromatography (HPLC) is the most widely used separation method for quantifying phenolic compounds [15,16]. The conditions include mainly the use of C18 reverse phase columns and a diode array and a fluorescence detector. Aqueous solutions and acetonitrile or methanol are usually the mobile phases. Notwithstanding, there are HPLC validated method*s* to quantify compounds similar to flavanones assayed [17]. The present job focused on the validation method of unpublished molecules prenylated flavanone **1** extracted from *E*. *platycarpa* and its derivatives **A**–**D**; considering the human skin as the principal biologic matrix. Moreover, this method was used to determine the concentration of prenylated flavanones in permeation and retention samples of ex vivo diffusional studies, using human skin and following bioanalytical guidelines to evaluate their intrinsic permeation, before they were analyzed in vivo as potential anti-inflammatory drugs candidates.

## 2. Materials and Methods

### 2.1. Chemicals and Reagents

The purified water used in all experiments was obtained from the MilliQ^®^ Plus System lab supply. All other chemical reagents used in this study were purchased from Fisher Scientific (Leicestershire, UK). The solvents were appropriately filtered through a 0.45 μm Millipore membrane filter (Merck, Darmstadt, Germany) and degassed in an ultrasonic bath for 20 min.

### 2.2. Extraction and Isolation of Plant Material

*E. platycarpa* leaves were collected in Tetipac, Guerrero, Mexico, and identified by Prof. Ramiro Cruz (Registration Number: Ramiro Cruz 1325 from the Sciences Faculty Herbarium Facilities of the Autonomous University of the State of Morelos). The leaves were dried at room temperature, then pulverized and extracted by three consecutive macerations with methanol at room temperature (100g of dried vegetal material per 1000 mL methanol). The extraction solvent was removed under reduced pressure. Next, the prenylated flavanone **1** was isolated by column chromatography at reduced pressure. Finally, it was purified and characterized by direct thin-layer chromatography (TLC) comparison with original samples available in the laboratory. The product was a yellow powder precipitate with a melting point of 200.2 °C. The compound obtained was characterized by comparison with previously published melting point data and with ^1^H-NMR results [9].

### 2.3. Semi-synthesis from Natural Prenylated Flavanone

Each prenylated flavanone was prepared following the method as previously reported [13] getting (2*S*)-5,7-bis(acetyloxy)-6-(3-methyl-2-buten-1-yl)-2-phenyl-2,3-dihydro-4H-1-Benzopyran-4-one **A**; (2*S*)-5-hydroxy-7-methoxy-6-(3-methyl-2-buten-1-yl)-2-phenyl-2,3-dihydro-4H-1-Benzopyran-4-one **B**; (8*S*)-5-hydroxy-2,2-dimethyl-8-phenyl-3,4,7,8-tetrahydro-2H,6H-Benzo[1,2-b:5,4-bˈ]dipyran-6-one **C**; and (8*S*)-5-hydroxy-2,2-dimethyl-8-phenyl-7,8-dihydro-2H,6H-Benzo[1,2-b:5,4-bˈ]dipyran-6-one **D**.

### 2.4. Chromatographic Operating Conditions

The HPLC system consisted of a Waters 515 HPLC pump, a 717 Plus autosampler, and a dual λ absorbance UV-vis 2487 detector (Waters, Milford, MA, USA). The analytical column was Atlantis^®^ C18 5 μm 250 mm × 4.6 mm, Waters. The analyte separation was performed with 10 μL sample injection volume. The separations were done in isocratic mode at room temperature. The mobile phase with a flow rate of 1 mL/min comprised of W-water and AcN-acetonitrile (%W: %AcN) with a different composition for each prenylated flavanone studied: **1** (30:70), **A** (20:80), **B** (40:60), **C** (20:80) and **D** (10:90). The detection wavelengths determined by spectrum scan were 300 nm for **1**, **B**, **C**, **D**, and 320 nm for **A**. The Peak area was used to quantify each analyte.

### 2.5. Ex Vivo Human Skin Permeation

A blank sample (ethanol: water; 70:30; v/v) was used as a negative control and the samples of prenylated flavanones (**1**, **A**, **B**, **C**, and **D**) with a concentration of 200 μg/mL were permeated through human skin membrane in the receptor compartment of the Franz diffusion cells (FDC 400, Crown Glass, Somerville, NY, USA), with a diffusion area of 2.54 cm^2^. Human skin from abdominal plastic surgery of healthy patients was used as a permeation membrane. The skin was cut into 400 μm thickness and placed between the donor, and the receptor compartment of the Franz diffusion cells, avoiding the formation of bubbles [18]. The flavanone samples **1**, **A**, **B**, **C**, and **D** (300 μL) were applied to the donor compartment and the receptor compartment was filled with ethanol: water (70:30) solution. The receptor compartment was kept at 32 ± 1 °C. Twenty-four h after the application of the tests, 300 μL aliquots were collected from the receptor side. Sink conditions were always followed. The flavanones amount permeated (Q) through human skin were determined by HPLC analysis described in Section 2.4.

### 2.6. Prenylated Flavanone Extraction

At the end of the ex vivo human skin permeation study, the flavanones amount*s* remaining in the skin were quantified by calculating the flavanone amount extracted from the skin to the flavanone amount added. For this purpose, the skin was removed from the Franz cells, cleaned with gauze soaked in a 0.05% solution of dodecyl sulfate and washed with distilled water. The permeated areas of the skin were then excised and weighed. The flavanone contained in the skin was extracted with ethanol: water (70:30) mixture under sonication (20 min) in an ultrasonic bath. The resulting solutions were measured with HPLC, quantifying the flavanone amount retained in the skin in micrograms of prenylated flavanone per grams of skin and per area unit μg/g_skin_.cm^2^).

### 2.7. Recovery from Human Skin Tissues and Prenylated Flavanone Retained

The accuracy of the extraction was evaluated by adding 1 mL of each prenylated flavanone solution (200 μg/mL) to their corresponding vials containing approximately 100 mg of human skin. These vials remained for 24 h at 32 °C to simulate the permeation conditions experiments. This experiment was conducted in triplicate. After the time of the study, the skin was submitted to drug extraction, as described in Section 2.6. The initial solutions and the eluates from each assay were collected and analyzed with HPLC. The differences obtained between the initial flavanone amount in the solution and the final flavanone amount in the collected solutions after 24 h were considered to be the value of the respective flavanone amount bound to tissue. Recovery percentage was calculated comparing the corresponding drug extraction results with the flavanone amount bound to the tissue [19]. A comparison of the amount of prenylated flavanone extracted and the recovery percentage was made in order to find out the real amount of flavanone retained in the skin.

### 2.8. Analytical Method Validation

The method was validated according to the International Conference on Harmonization guidelines (ICH) [20,21] for linearity, the limit of detection (LOD), the limit of quantification (LOQ), accuracy, and precision. Calibration curves were analyzed in two ranges; from 200 to 12.5 μg/mL in a high concentration level, and from 12.5 to 1.56 μg/mL in a low concentration level.

#### 2.8.1. Standard Solutions for Calibration Curves

Standard stock solutions of each compound (**1**, **A**, **B**, **C**, and **D**) were prepared daily by dissolving the appropriate amount of each analyte in ethanol: water (7:3; v/v) to obtain a final concentration of (200 μg/mL). The working solutions were elaborated by the dilution of appropriate aliquots of the stock solutions with the diluting solvent to reach the concentration ranges 1.56, 3.12, 6.25, 12.5, 25, 50, 100 μg/mL.

#### 2.8.2. Linearity

The linearity was evaluated by a one-way analysis of variance (ANOVA) test to compare peak areas versus nominal concentrations of each standard [22]. Differences were considered statistically significant when *p* ˂ 0.05. The least-square linear regression analysis and mathematical determinations were performed by Prism, V 5.0 software (Graph Pad Software Inc., San Diego, CA, USA).

#### 2.8.3. Limit of Detection and Limit of Quantification

The limit of detection (LOD) and the limit of quantitation (LOQ) for each analyte (**1**, **A**, **B**, **C**, and **D**) were calculated based on the standard deviation of the response and the slope of the calibration curve, generated from six replicate analysis applying the formula 1 [23]:

(1) $$LOD\;or\;LOQ=k\frac{SD_{Sa}}{S_{b}}$$

where *k* is the factor related to the level of confidence (*k* = 3.3 and 10 for LOD and LOQ respectively), SD_Sa_ is the standard deviation of the intercept, and S_b_ is the slope.

#### 2.8.4. Repeatability, Accuracy, and Precision

The instrumental repeatability was assayed by analyzing the concentration sample of 200 μg/mL for each flavanone (**1**, **A**, **B**, **C**, and **D**) repeatedly seven times, consecutively. The accuracy and precision were investigated by measuring samples in three concentrations 1.56, 12.5, and 200 μg/mL [24]. The inter-day test was conducted by analyzing each analyte (**1**, **A**, **B**, **C**, and **D**) with each of the three concentration levels mentioned before, once a day for six consecutive days. The accuracy was expressed as a relative error (RE%). The precision was defined as the relative standard deviation (RSD%) of the measurement. The method is considered accurate and precise if RE% and RSD%, respectively, are within ±15%.

#### 2.8.5. Specificity

Specificity is defined as the ability of the method to distinguish the analyte from all other substances present in the sample. This can be proven by comparing the analyte chromatographic retention time in extracted matrix samples and with its retention time in at least one reference solution [19,25]. To test the specificity of the analytical method, the ex vivo human permeation procedure described in Section 2.5 was followed. The blank sample peaks should not appear at the same retention times of the prenylated flavanones.

## 3. Results and Discussions

Due to the fact of the biological properties of prenylated flavanones, it is of utmost importance to count on analytical method validation in order to promote future studies. HPLC is highly sensitive in the determination of small quantities of natural molecules in biopharmaceutical studies based on previous studies [22].

### 3.1. Analytical Method Validation

#### 3.1.1. Linearity

The linearity of the analytical method is the capability over a range of data to obtain proportional results. The applied HPLC method for the flavanone’s quantification (**1**, **A**, **B**, **C,** and **D**) showed satisfactory linearity in the tested concentrations. In order to have a better mathematical analysis, the linearity was evaluated in two concentration ranges: from 200 to 12.5 μg/mL and from 12.5 to 1.56 μg/mL. Two linear calibration curves were fitted for each flavanone. The R^2^ value for each analyte was found above 0.999 for all studied flavanones, indicating the linear relationship between the analyte concentration and the peak area. No statistical differences were found (*p* ˃ 0.05) after the *ANOVA* test of the calibration curves of each flavanone **1**, **A**, **B**, **C,** and **D** with *p* values for each two levels of 0.12 and 0.08, 0.93 and 0.08, 0.38 and 0.47, 0.63 and 0.56, and 0.53 and 0.46, respectively (see Table 1).

#### 3.1.2. Limit of Detection and Limit of Quantification

LODs and LOQs for all the investigated flavanones were calculated using the response standard deviation and the calibration curve slope of 12.5 to 1.56 μg/mL for each flavanone, described in Section 2.8.3. The values of LODs and LOQs for each flavanone are listed in Table 1. These results indicate that the method is sensitive enough for flavanones determination in the range of 1.56 to 200 μg/mL.

#### 3.1.3. Repeatability, Accuracy, and Precision

Precision and accuracy values were obtained from sample analyses of the 1.56, 12.5, and 200 μg/mL flavanones concentrations (**1**, **A**, **B**, **C,** and **D**). The inter-day precision and accuracy were calculated after analyzing the samples on six different days. The results are reported in Table 1. Both parameters were lower than the 15 % limit value in EMA (European Medicines Agency) guidelines. These results suggest that the proposed method has satisfactory accuracy and precision. Repeatability studies of the instrumental system showed RSD % not greater than 0.6% for all flavanones.

#### 3.1.4. Specificity

The analytical methodology was implemented for the flavanone’s quantitation (**1**, **A**, **B**, **C,** and **D**) in skin permeation studies. In order to show the specificity, 300 μL of the flavanones at 200 μg/mL concentration was permeated into human skin using Franz cells. The amount of each flavanone normalized by the surface (Q) in the receptor compartment during percutaneous permeation experiments (*n* = 3) is indicated in Table 2. Percentages of permeation were calculated, accounting for the experimental flavanone content of each assay. At the end of each experiment, skin samples were removed from the diffusion cell, flavanones amount retained (Q_ret_) were quantified as previously described and expressed in micrograms of prenylated flavanone per grams of skin and per area unit (μg/g_skin_.cm^2^) in Table 2. As shown in Table 2, the prenylated flavanone **1** is the one which most permeates, followed by **D** and **C.** In the case of **A** and **B**, Q was detectable but not quantifiable because their values were below LOQ determinate before (see Table 1). On the contrary, **A** and **B** are found retained in the skin in a greater amount, so we can infer that it is possible that these molecules have greater interactions with the different skin components due to their physicochemical characteristics. Therefore, they have prevented permeation as **1, C**, and **D** did too.

**Figure 2:**
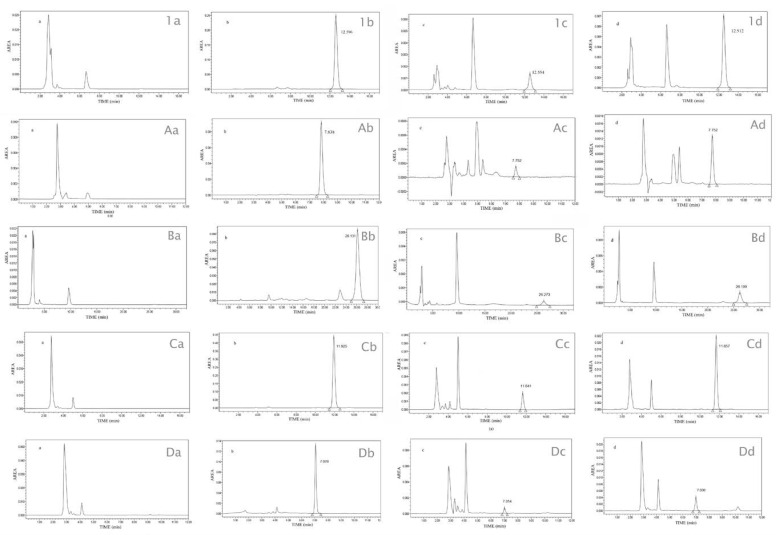
HPLC chromatograms of prenylated flavanones **1**, **A**–**D** classified with sub-index a, b, c, and d. _a_ correspond to: Blank sample (ethanol: water, 70:30) permeated at chromatographic conditions of each prenylated flavanone; _b_ correspond to prenylated flavanone standard; _c_ correspond to prenylated flavanone from receptor compartment Franz diffusion cells; and, _d_ correspond to prenylated flavanone extracted from human skin after permeation study.

The chromatograms showed the absence of interference of any other peak corresponding to each flavanone (Figure 2: 1_a_, 1_b_; A_a_, A_b_; B_a_, B_b_; C_a_, C_b_; D_a_, D_b_). Furthermore, no interference from the human skin components assays were observed during the analysis in the ex vivo permeation studies (Figure 2: 1_c_, 1_d_; A_c_, A_d_; B_c_, B_d_; C_c_, C_d_; D_c_, D_d_).

#### 3.1.5. Recovery

Flavanone extraction was done, as described in Section 2.6. Recovery was calculated comparing the corresponding extraction result with the amount of flavanone bound to the skin. The results were reported as the mean value of the percentage between the amount of flavanone in each sample and the weight of the skin sample (see in Table 2). The aforementioned results show the real quantity for each prenylated flavanone that can be recovered using the extraction method previously described. Thus, the exact quantity of flavanone retained can be known, and this quantity is responsible for the exercising of the biological effect.

## 4. Conclusions

The results in the present research describe a liquid chromatographic method validation for the analysis of prenylated flavanones **1**, **A**–**D** using UV-VIS detection. According to the data obtained, the method developed is linear, accurate, and precise. In addition, the method can be used in the quantification of prenylated flavanones samples from permeation and retention studies in human skin. Finally, the method is selective since the chromatograms allowed the observer to identify the signal for each prenylated flavanone without interference. We can conclude that this method is suitable for further analysis of prenylated flavanones **1**, **A**–**D** in biological systems, and for other biopharmaceutical studies.

## Figures and Tables

**Table 1:** Linearity (expressed in R^2^ and *p* with two ranges, one by row), Precision, Accuracy (calculated at maximum, medium, and minimum concentration values), and Repeatability of the HPLC Method for the determination of flavanones.

Compound	Linearity		LOD	LOQ	Accuracy	Precision	R.I.S
	R^2^	*p*			RE (%)	RSD (%)	RSD (%)
	200–12.5 (μg/mL)		Mean (μg /mL)		200 (μg /mL)		200 (μg /mL)
	12.5–1.56 (μg /mL)		± SD (μg /mL)		12.5 (μg /mL)		
					1.56 (μg /mL)		
**1**	0.9998	0.12	0.51 ± 0.13	1.53 ± 0.38	−0.23	0.20	0.36
	0.9991	0.08			0.59	0.24	
					11.02	2.63	
**A**	0.9997	0.93	0.28 ± 0.10	0.84 ± 0.29	−0.09	0.09	0.54
	0.9997	0.08			0.63	0.21	
					1.48	0.81	
**B**	0.9998	0.38	0.49 ± 0.12	1.48 ± 0.36	0.46	0.27	0.43
	0.9990	0.47			−0.13	0.36	
					−7.91	2.92	
**C**	0.9999	0.63	0.48 ± 0.43	1.45 ± 1.30	−0.19	0.29	0.37
	0.9996	0.56			0.38	0.07	
					4.15	1.12	
**D**	0.9999	0.53	0.30 ± 0.08	0.91 ± 0.24	−0.01	0.21	0.32
	0.9997	0.46			0.17	0.27	
					3.74	0.42	

LOD = limit of detection; LOQ = limit of quantitation; RE =relative error; RSD = relative standard deviation; and R.I.S = Repeatability of Instrumental System.

**Table 2:** Results of the permeation studies expressed by mean and SD (*n* = 3).

Compound	24 h Permeated Amount	Degree Of Permeation	Recovery	Skin Retention
	Q (μg/cm^2^)	(%)	(%)	Q_ret_ (μg/g.cm^2^)
**1**	1.29 ± 0.12	2.15	46.20 ± 6.46	50.22 ± 7.51
**A**	NQ	NQ	0.38 ± 0.05	321.52 ± 45.23
**B**	NQ	NQ	3.43 ± 0.5	381.75 ± 57.26
**C**	0.75 ± 0.07	1.26	NQ	23.78 ± 5.46 *
**D**	0.91 ± 0.08	1.52	38.1 ± 5.23	116.14 ± 17.24

^1^ NQ = non quantifiable; value below LOQ, * skin extracted not corrected by percentage recovery.
